# Module Development: Psychological-Based Prehabilitation for Breast Cancer Patients and their Family Caregivers

**DOI:** 10.1192/j.eurpsy.2025.1791

**Published:** 2025-08-26

**Authors:** H. S. Saw, Y. Y. Chen

**Affiliations:** 1 Department of Psychological Medicine, Faculty of Medicine and Health Sciences, University of Malaysia, Kota Samarahan, Malaysia

## Abstract

**Introduction:**

Cancer prehabilitation has emerged as a pivotal strategy for the augmentation of patients’ comprehensive well-being, encompassing physical, psychological, and functional aspects while improving their quality of life. The profound impact of breast cancer on patients and their family caregivers emphasizes the critical need for interventions addressing their holistic well-being.

**Objectives:**

In this portion of the study, researchers intend to develop two psychological-based prehabilitation modules within a larger investigation targeting to address the quality of life as well as relationship quality among breast cancer patients and their family caregivers. The deliberate involvement of the family caregiver proposed here is based on the belief that the well-being of an individual is interconnected with his/her community.

**Methods:**

Guided by the Allostatic Model and Systems Theory, these modules are tailored to mitigate the negative effects associated with breast cancer with a systemic perspective in mind. Leveraging on Sidek’s Module Development Model, the first module (PreCARE) targets breast cancer patients, while the second (PreCARE Plus) extends its focus to patients and their family caregivers. The content validity of these modules is rigorously assessed through consultation with six experts across mental health, oncology, rehabilitation, and radiology disciplines. The study aims to look at the aspects of content validity of the developed modules through the content validity index (CVI).

**Results:**

The CVI for both PreCARE and PreCARE Plus modules were high (above 0.83) with values of 0.92 and 0.93 respectively. The findings also yielded high S-CVI for both modules with values of 0.98 (PreCARE) and 0.96 (PreCARE Plus).

**Conclusions:**

These crafted psychological-based prehabilitation modules proposed to revolutionize oncological care in Malaysia by empowering healthcare professionals in the oncology setting to enhance the psychological and physical well-being of breast cancer patients and their family caregivers. Their potential impact underscores the importance of integrating systemic-inspired interventions into comprehensive cancer care paradigms.

**Disclosure of Interest:**

None Declared

